# Effects of Acute Moderate Hypoxia versus Normoxia on Metabolic and Cardiac Function and Skeletal Muscle Oxygenation during Endurance Exercise at the Same Heart Rate Level

**DOI:** 10.3390/metabo12100975

**Published:** 2022-10-15

**Authors:** Hun-Young Park, Won-Sang Jung, Sung-Woo Kim, Jisu Seo, Yerin Sun, Jae-Ho Choi, Jisu Kim, Kiwon Lim

**Affiliations:** 1 Physical Activity and Performance Institute, Konkuk University, Seoul 05029, Korea; 2 Department of Sports Medicine and Science, Graduate School, Konkuk University, Seoul 05029, Korea; 3 Department of Physical Education, Konkuk University, Seoul 05029, Korea

**Keywords:** metabolic function, cardiac function, skeletal muscle oxygenation, endurance exercise, same heart rate level, hypoxia

## Abstract

This study aimed to investigate the effects of acute moderate hypoxia (HYP), compared with those of normoxia (NORM), during endurance exercise with the same HR level on metabolic function, skeletal muscle oxygenation, and cardiac function. Twelve healthy men (aged 25.1 ± 2.3 years) completed 30 min of endurance exercise using a cycle ergometer with the same HR level (136.5 ± 1.5 bpm) corresponding to 70% maximal heart rate (HRmax) under NORM (760 mmHg) and HYP (526 mmHg, simulated 3000 m altitude) after a 30 min exposure in the respective environments on different days, in random order. Exercise load, rating of perceived exertion (RPE), metabolic function (saturation of percutaneous oxygen; SpO_2_, minute ventilation; oxygen uptake; VO_2_, carbon dioxide excretion; respiratory exchange ratio; RER, and oxygen pulse), skeletal muscle oxygen profiles (oxyhemoglobin, oxhb, deoxyhemoglobin, dxhb, total hemoglobin, and tissue oxygenation index; StO_2_), and cardiac function (heart rate, stroke volume, cardiac output, end-diastolic volume, end-systolic volume, and ejection fraction) were measured during endurance exercise. HYP showed a lower exercise load with the same RPE during exercise than did NORM. In addition, HYP showed a lower SpO_2_, VO_2_, oxygen pulse, oxhb, and StO_2_, and a higher RER and dxhb during exercise than NORM. We found that HYP showed lower exercise load and VO_2_ at the same RPE than NORM and also confirmed a higher anaerobic metabolism and oxygen inflow into skeletal muscle tissue due to the limitation of oxygen delivery capacity.

## 1. Introduction

Hypoxia refers to a state in which both atmospheric and oxygen pressures decrease as the pressure and amount of air decrease, such as in a natural high-altitude environment [1].

The primary responses of the respiratory and circulatory systems under hypoxia are caused by lower oxygen content in blood due to lower fraction of inspiratory oxygen, which is related to decreased arterial oxygen content and saturation (SaO_2_) and arterial-venous oxygen difference via atmospheric oxygen partial pressure [2]. Endurance exercise under hypoxia reduces SaO_2_, decreasing the ability of activated muscles to deliver and utilize oxygen [3]. In addition, exercise under hypoxia augments the relative exercise intensity to increase the heart rate (HR) for oxygen delivery to tissues and increases the dependence on anaerobic metabolism via carbohydrate substrate mobilization with high oxygen utilization efficiency and blood lactate levels [4,5,6,7]. In other words, exercise under hypoxia at the same exercise load imposes a relative burden on individuals via decreased SaO_2_ and increased minute ventilation (VE), oxygen uptake (VO_2_), and blood lactate level during submaximal exercise, along with decreased maximal oxygen uptake (VO_2_max) and peak power output [5,8,9].

In addition, exercise under hypoxia has been shown to have some adverse effects on skeletal muscle oxygen profiles [10,11,12,13]. Acute exposure to hypoxia induces systemic hypoxia, which can compromise skeletal muscle oxygenation and subsequently, skeletal muscle ability [14,15]. These acute responses of skeletal muscle oxygenation under hypoxia induce attenuated aerobic exercise capacity and VO_2_max, as well as changes in metabolic function [9,13]. Regarding cardiac function, exercise corresponding to the same exercise load under hypoxia induces increased HR and cardiac output (CO) to compensate for the decrease in oxygen delivery and utilization capacities and the activated sympathetic nervous system [16,17]. However, previous studies have reported inconsistent results regarding cardiac function parameters, such as stroke volume (SV), end-diastolic volume (EDV), end-systolic volume (ESV), and ejection fraction (EF) during exercise under hypoxia [16,17,18,19,20].

The majority of previous studies comparing the acute response during exercise under hypoxia and normoxia examined the changes in various physiological parameters in response to the same load or relative intensity during submaximal exercise and maximal exercise [3,4,5,6,7,8,10,12,13,17,20]. However, studies on metabolic function, skeletal muscle oxygenation, and cardiac function responses during exercise with the same HR level under hypoxia and normoxia are limited [21]. Usually, the general population performs exercise training based on HR in daily life for health promotion or disease prevention, and it is an important task to confirm the difference in physiological responses during exercise at the same HR level under normoxia and hypoxia [22]. Therefore, we conducted a randomized crossover trial in healthy men to compare the effects of acute moderate hypoxia (526 mmHg, simulated 3000 m) and normoxia on metabolic and cardiac function, as well as skeletal muscle oxygenation, during endurance exercise at the same HR level. We hypothesized that endurance exercise at the same HR level under moderate hypoxia would produce greater responses in metabolic function, skeletal muscle oxygenation, and cardiac function than it did under normoxia in healthy men.

## 2. Materials and Methods

### 2.1. Participants

This study included 12 healthy men (age, 25.1 ± 2.3 years; height, 179.9 ± 4.4 cm; weight, 83.6 ± 13.3 kg; body mass index, 25.8 ± 3.6 kg/m^2^; fat-free mass, 67.7 ± 6.1 kg; fat mass, 15.9 ± 9.2 kg; percent body fat, 18.2 ± 7.5%) who were nonsmokers and had no history of musculoskeletal, cardiovascular, or pulmonary diseases and did not participate in any planned exercise program and did not consume any dietary supplements in the previous 6 months. All participants were also identified as healthy people via Physical Activity Readiness Questionnaire for Everyone [23]. However, the amount of physical activity and nutritional intake of the participants was not investigated.

**Figure 1:**
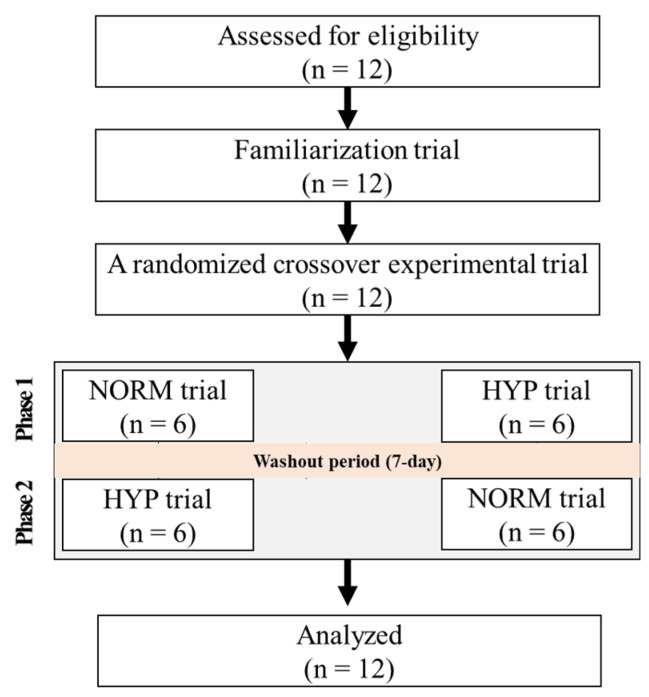
The flow diagram of the consolidated standards of reporting the trial. NORM: endurance exercise under normoxia; HYP: endurance exercise under moderate hypoxia (526 mmHg, simulated 3000 m).

A flow diagram of the consolidated standards of the reporting trial is shown in Figure 1. All participants received information about the purpose and process of the study and provided informed consent prior to the start of the study. This study was approved by the Institutional Review Board of Konkuk University (7001355-201805-HR-241) in Korea and was conducted in accordance with the Declaration of Helsinki.

### 2.2. Study Design

**Figure 2:**
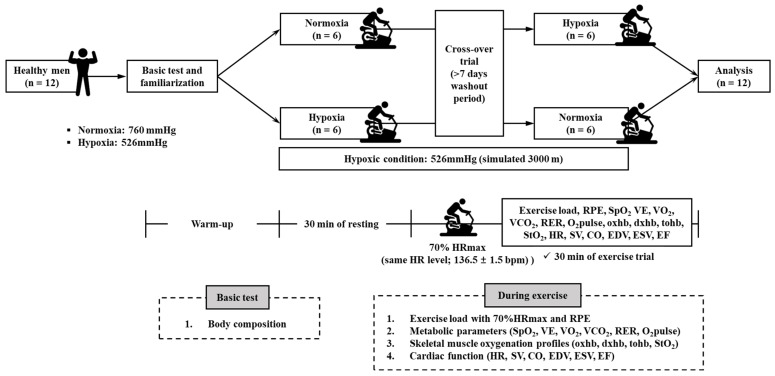
Study design. HRmax: maximal heart rate; RPE: rating of perceived exertion; SpO_2_: saturation of percutaneous oxygen; VE: minute ventilation; VO_2_: oxygen uptake; VCO_2_: carbon dioxide excretion; RER: respiratory exchange ratio, O_2_pulse: oxygen pulse; oxhb: oxygenated hemoglobin; dxhb: deoxygenated hemoglobin; tohb: total hemoglobin; StO_2_: tissue oxygenated saturation; HR: heart rate; SV: stroke volume; CO: cardiac output; EDV: end-diastolic volume; ESV: end-systolic volume, EF: ejection fraction.

The study design is illustrated in Figure 2. The participants visited the laboratory thrice during the experimental period. During the first visit, all participants fasted for more than 4 h, and their height and body composition were measured in the morning after stabilization. Subsequently, all the participants performed a familiarization trial with the exercise protocol. The exercise protocol consisted of 30 min of endurance exercise on a cycle ergometer (Aerobike 75XLII; Konami Corporation, Tokyo, Japan) at the same HR level equivalent to 70% maximal HR (70%HRmax) calculated using the Miyashita formula (male = 209 − 0.69 × age) [4]. The target HR of the participants, 70% HRmax, was 136.5 ± 1.5 bpm. During the second and third visits, the participants performed the experimental trials in a crossover randomized order under normoxia (760 mmHg, equivalent to sea level, NORM) and moderate hypoxia (526 mmHg, equivalent to an altitude of 3000 m, HYP). During 30 min of the endurance exercise session under each environmental condition, the following parameters were measured: exercise load and rating of perceived exertion (RPE), metabolic function (saturation of percutaneous oxygen; SpO_2_, VE, VO_2_, carbon dioxide excretion; VCO_2_, respiratory exchange ratio; RER, and oxygen pulse), skeletal muscle oxygenation (oxhb: oxygenated hemoglobin, dxhb: deoxygenated hemoglobin, tohb: total hemoglobin, and StO_2_: tissue oxygenated saturation), and cardiac function (HR, SV, CO, EDV, ESV, and CO). RPE and SpO_2_ were measured every 5 min, whereas the remaining parameters were measured every minute.

The environmental conditions used in the present study were simulated using a 9 m (width) × 7 m (length) × 3 m (height) environmental control chamber (NCTC-1, Nara control, Seoul, Republic of Korea) with the capacity to simulate hypoxic conditions for high altitudes (up to 609 mmHg, equivalent to an altitude of 6000 m). Temperature and humidity within the environmental chamber were maintained at 20 ± 2°C and 60 ± 2%, respectively, under all environmental conditions. The trials were spaced at least one week apart and performed at the same time of the day, and the order of the trials was randomized.

### 2.3. Measurement

Body composition (i.e., height, weight, fat-free mass, and percentage of body fat) was measured after fasting for more than 4 h using a bioelectrical impedance analysis device (Inbody 770; Inbody, Seoul, Korea).

The exercise load corresponding to 70%HRmax was automatically measured every minute using a cycle ergometer, and the RPE was measured every 5 min using a 6–20 Borg scale.

For the metabolic function, SpO_2_ was measured every 5 min, and the remaining parameters (VE, VO_2_, VCO_2_, RER, and oxygen pulse) were measured every minute. The SpO_2_ was measured using a Radical-7 pulse oximeter (Masimo, Irvine, CA, USA), and VE, VO_2_, VCO_2_, RER, and oxygen pulse were measured using the K5 auto metabolism analyzer (Cosmed, Rome, Italy) and using a breathing valve in the form of a facemask during 30 min of the endurance exercise.

Skeletal muscle oxygen, including oxhb, dxhb, tohb, and StO_2_, was measured every minute using an NIRS system for muscle tissue (Astem; Mizonokuchi, Japan) in the right vastus lateralis muscle. After attaching the NIRS probe to the right vastus lateralis muscle, 10–15 cm above the knee, data were recorded every 10 s from rest to the end of the 30 min endurance exercise, and an average value of 1 min was used.

Cardiac functions, including HR, SV, CO, EDV, ESV, and EF, were noninvasively assessed every minute using a thoracic bioelectrical impedance device (PhysioFlow PF-05; Paris, France) during 30 min of the endurance exercise.

### 2.4. Statistical Analysis

All statistical analyses were performed using SPSS version 25.0 (IBM Corp., Armonk, NY, USA) for Windows. Data are presented as the mean ± standard deviation (S.D.). To determine the sample size, we focused on identifying meaningful differences in SpO_2_ during submaximal exercise, as previously suggested [3]. A priori power analysis, which was performed using G-power, indicated that a minimum sample size of 10 participants would be required to provide 90% power at an α level of 0.05. Anticipating a dropout rate of >10%, we aimed for a starting sample size of 12 participants. The normality of the distribution of all outcome variables was verified using the Shapiro–Wilk W-test prior to parametric tests. Two-way analysis (trial × time) of variance (ANOVA) with repeated measures was used to assess the presence of interactions (environment × exercise) and main effects (trial or time). When the ANOVA revealed a significant interaction or main effect within the environment, a Bonferroni post hoc test was used to identify within-environment differences. The level of significance was set a priori at *p* < 0.05.

## 3. Results

### 3.1. Exercise Load and RPE

**Figure 3:**
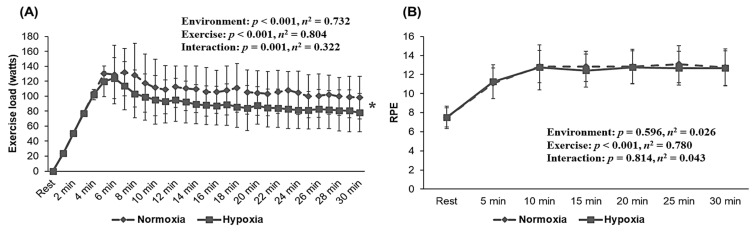
Changes in exercise load and RPE during 30 min of endurance exercise at the same HR level (136.5 ± 1.5 bpm) under normoxia (760 mmHg) and hypoxia (526 mmHg, simulated 3000 m). (**A**) Exercise load, (**B**) RPE. HRmax: maximal heart rate; RER: rating of perceived exertion. The bars indicate the mean ± S.D. *: Significant difference within the environmental condition (normoxia vs. hypoxia) during 30 min of endurance exercise.

As shown in Figure 3, exercise load presented a significant interaction (*p* = 0.001, *η*^2^ = 0.322) and main effect within the environment (*p* < 0.001, *η*^2^ = 0.732), whereas RPE showed no significant interaction or main effect within the environment. Post hoc analysis indicated that HYP showed a lower exercise load during endurance exercise at the same HR level as that of NORM (*p* < 0.05).

### 3.2. Metabolic Function

**Figure 4:**
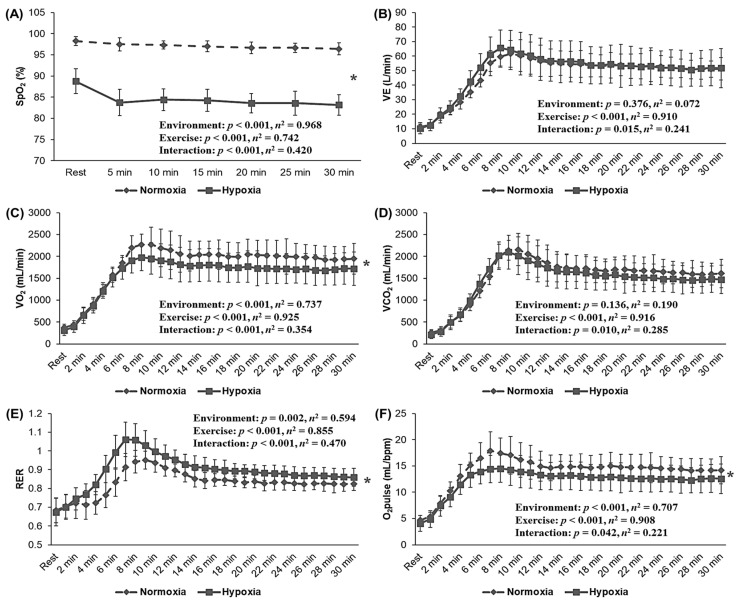
Changes in metabolic function during 30 min of the endurance exercise at the same HR level (136.5 ± 1.5 bpm) under normoxia (760 mmHg) and hypoxia (526 mmHg, simulated 3000 m). (**A**) SpO_2_, (**B**) VE, (**C**) VO_2_, (**D**) VCO_2_, (**E**) RER, (**F**) O_2_ pulse. HRmax: maximal heart rate; SpO_2_: saturation of percutaneous oxygen; VE: minute ventilation; VO_2_: oxygen uptake; VCO_2_: carbon dioxide excretion; RER: respiratory exchange ratio, O_2_pulse: oxygen pulse. The bars indicate the mean ± S.D. *: Significant difference within the environmental condition (normoxia vs. hypoxia) during 30 min of the endurance exercise.

Figure 4 illustrates the metabolic function during 30 min of the endurance exercise. All metabolic function parameters, including SpO_2_ (*p* < 0.001, *η*^2^ = 0.420), VE (*p* = 0.015, *η*^2^ = 0.241), VO_2_ (*p* < 0.001, *η*^2^ = 0.354), VCO_2_ (*p* = 0.010, *η*^2^ = 0.285), RER (*p* < 0.001, *η*^2^ = 0.470), and oxygen pulse (*p* = 0.042, *η*^2^ = 0.221), showed a significant interaction, and SpO_2_ (*p* < 0.001, *η*^2^ = 0.968), VO_2_ (*p* < 0.001, *η*^2^ = 0.737), RER (*p* = 0.002, *η*^2^ = 0.594), and oxygen pulse (*p* < 0.001, *η*^2^ = 0.707) also showed a significant main effect within the environment. Post hoc analysis indicated that endurance exercise under HYP showed lower SpO_2_, VO_2_, and oxygen pulse and higher RER at the same HR level than it did under NORM (*p* < 0.05).

### 3.3. Skeletal Muscle Oxygenation

**Figure 5:**
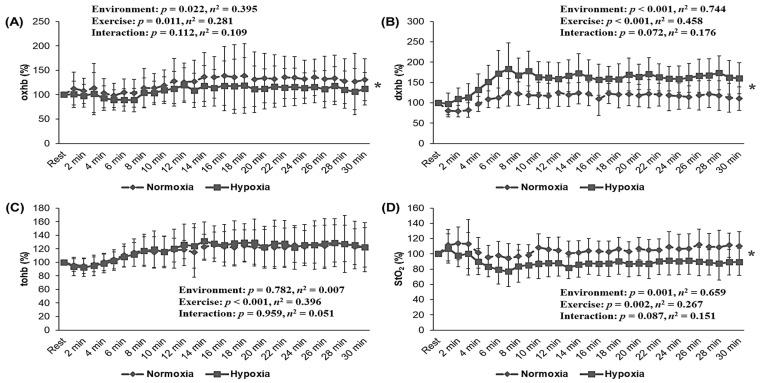
Changes in skeletal muscle oxygenation during 30 min of endurance exercise at the same relative exercise intensity (70%HRmax) under normoxia (760 mmHg) and hypoxia (526 mmHg, simulated 3000 m). (**A**) oxhb, (**B**) dxhb, (**C**) tohb, (**D**) StO_2_. HRmax: maximal heart rate; oxhb: oxygenated hemoglobin; dxhb: deoxygenated hemoglobin; tohb: total hemoglobin; StO_2_: tissue oxygenated saturation. The bars indicate the mean ± S.D. *: Significant difference within the environmental condition (normoxia vs. hypoxia) during 30 min of the endurance exercise.

In skeletal muscle oxygen profiles, as shown in Figure 5, oxhb (*p* = 0.022, *η*^2^ = 0.395), dxhb (*p* < 0.001, *η*^2^ = 0.744), and StO_2_ (*p* = 0.001, *η*^2^ = 0.659) presented a significant main effect within the environment, whereas tohb showed no significant interaction or main effect within the environment. Post hoc analysis indicated that HYP showed lower oxhb and tohb and higher dxhb during endurance exercise with the same HR level as NORM (*p* < 0.05).

### 3.4. Cardiac Function

**Figure 6:**
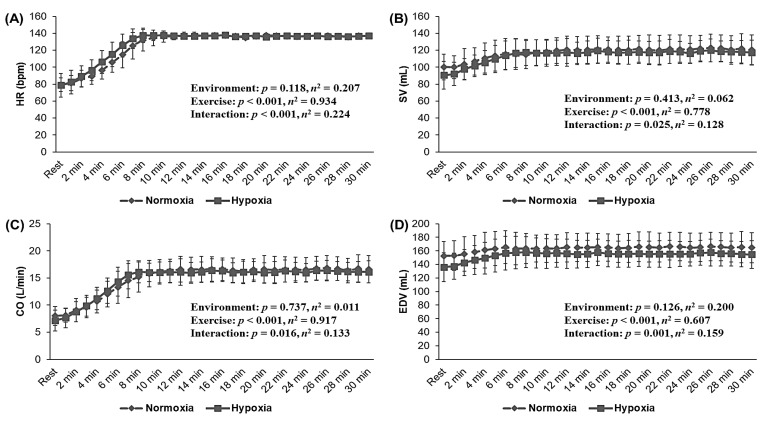

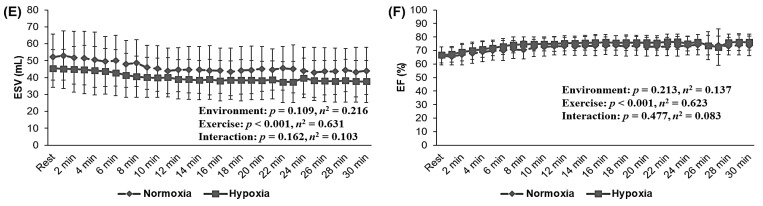
Changes in cardiac function during 30 min of the endurance exercise at the same relative exercise intensity (70%HRmax) under normoxia (760 mmHg) and hypoxia (526 mmHg, simulated 3000 m). (**A**) HR, (**B**) SV, (**C**) CO, (**D**) EDV, (**E**) ESV, (**F**) EF. HRmax: maximal heart rate; HR: heart rate; SV: stroke volume; CO: cardiac output; EDV: end-diastolic volume; ESV: end-systolic volume, EF: ejection fraction. The bars indicate the mean ± S.D.

Figure 6 illustrates the cardiac function during 30 min of the endurance exercise. All cardiac function parameters, including HR, SV, CO, EDV, ESV, and EF, showed no significant interaction or main effect within the environment. In other words, no significant difference was observed between NORM and HYP during endurance exercise at the same HR level.

## 4. Discussion

We hypothesized that endurance exercise at the same HR level under moderate hypoxia would induce greater responses in metabolic function and skeletal muscle oxygenation than under normoxia in healthy men. Consistent with this hypothesis, HYP showed a greater response in metabolic function (e.g., lower SpO_2_, VO_2_, and oxygen pulse and higher RER) and skeletal muscle oxygenation (e.g., lower oxhb and tohb, and higher dxhb) with a lower exercise load during endurance exercise with the same HR level. However, no significant changes were observed in cardiac function during endurance exercise at the same HR level. These results suggest that exercise at the same HR provides the participants with a lower exercise load, but results in greater metabolic stimulation, indicating that it can enhance metabolic utility during long-term exercise [24].

In metabolic function, the lower atmospheric partial pressure of oxygen under hypoxia decreases SpO_2_ and the arteriovenous oxygen difference and worsens endurance exercise performance [3,4,5,6]. Acute exposure to hypoxia also reduces the oxygen content in blood and the muscle’s ability to use oxygen, which increases oxygen demand, resulting in a decrease in VO_2_ during submaximal exercise at the same relative intensity and VO_2_max [3,9,17]. Endurance exercise under hypoxia causes greater metabolic stress (e.g., higher blood lactate level and lower S_p_O_2_, bicarbonate ion, and blood pH) than under normoxic conditions, despite lower mechanical stress (i.e., lower exercise load) [4,5,6]. In a hypoxic environment, increased metabolic responses via a decrease in oxygen partial pressure may decrease aerobic energy metabolism by increasing cardiometabolic responses and anaerobic energy metabolism [25,26]. In our study, we confirmed that HYP showed greater metabolic function responses (e.g., lower SpO_2_, VO_2_, and oxygen pulse and higher RER) with a lower exercise load during endurance exercise at the same relative exercise intensity.

Consistent with our results, Sumi et al. [6] who investigated metabolic function in response to exercise under moderate hypoxia (fraction of inspired oxygen (FiO_2_) = 14.5%, simulated 3000 m) vs. normoxia among endurance athletes reported that endurance exercise under hypoxia induced greater metabolic responses, such as increased blood lactate level and decreased blood pH and bicarbonate ion concentration, compared with those with the same exercise in normoxia, despite lower mechanical stress. Nam and Park [4] investigated the metabolic responses at rest and after exercise in healthy men under normoxia and hypoxia. They confirmed that endurance exercise with the same exercise load under hypoxia induced a greater difference in metabolic stress, including blood glucose and lactate levels, SpO_2_, pH, and bicarbonate ion concentration, compared with exercise under normoxia. However, Park et al. [3] compared the effects of metabolic responses to submaximal exercise under hypoxic and normoxic conditions. They confirmed that endurance exercise under hypoxia with the same exercise load (i.e., exercise load at 60%VO_2_max under normoxia) decreased SpO_2_ and increased blood lactate levels. However, unlike our results, the authors reported no significant responses in VE, VO_2_, RER, and VCO_2_ during endurance exercise under hypoxia vs. normoxia. These differences seem to be due to differences in exercise intensity (i.e., absolute intensity vs. relative intensity) during endurance exercise under hypoxia and normoxia. The main finding of our study was that HYP showed a higher RER and a lower oxygen pulse despite a lower VO_2_ due to a lower exercise load (watts) at the same relative intensity during endurance exercise.

Skeletal muscle oxygenation, measured using NIRS, reflects the dynamic balance between local oxygen supply by microcirculation and oxygen consumption by the mitochondria [27]. Endurance exercise under hypoxia decreases skeletal muscle oxygenation, such as decreased oxhb and StO_2_, and increased dxhb compared with that under normoxia. Skeletal muscle oxygenation during endurance exercise under hypoxia can decrease due to increased skeletal muscle extraction and a reduction in oxygen supply to the skeletal muscle tissue compared with that under normoxia [11,12,13,28]. Therefore, we examined changes in skeletal muscle oxygenation during endurance exercise at the same relative exercise intensity under hypoxia and normoxia. We confirmed that HYP showed lower oxhb and tohb and higher dxhb during endurance exercise at the same relative exercise intensity as that of NORM. In other words, endurance exercise under hypoxia results in a lower oxhb and StO_2_ and a higher dxhb to deliver more oxygen to active muscle tissue because oxygen delivery capacity is reduced due to the reduction of SpO_2_ and oxygen pulse, even though it indicates a lower exercise load compared with that under normoxia. These results appear to be important findings relating to exercise in hypoxia and skeletal muscle oxygenation levels [11].

Regarding cardiac function, our study did not show significant changes in cardiac function during endurance exercise under hypoxia, at the same relative exercise intensity compared with that under normoxia. Exercise under hypoxia increases HR to compensate for decreased oxygen delivery and utilization capacity and activates the sympathetic nervous system [16,17]. The accumulation of metabolites (e.g., lactate and hydrogen ions) during exercise under hypoxia activates the afferent nerve impulses of metabolic receptors “Group IV,” thereby activating the sympathetic nervous system [17,28]. In addition, endurance exercise under hypoxia with the same exercise load increases ventricular contractility, SV, and CO by stimulating β-adrenergic receptors [29,30]. These phenomena may indicate that activation of the sympathetic nervous system and increased catecholamine levels in the blood are triggered by increased calcium ions in cardiac muscle cells during exercise under hypoxia [17,29,30]. However, unlike previous studies, our study confirmed no significant difference in cardiac function, such as HR, SV, CO, EDV, ESV, and EF, between NORM and HYP during endurance exercise at the same relative exercise intensity. These differences in cardiac function appear to be due to the fact that the exercise intensity was set to the same relative intensity (e.g., same HR during exercise) in the present study. In other words, we confirmed that endurance exercise under HYP showed a lower exercise load (e.g., a lower cycle ergometer load) than it did under NORM; therefore, there was no significant difference in cardiac function between both trials.

## 5. Limitations

In the present study, the normality of distribution of all outcome parameters was verified using the Shapiro–Wilk W-test prior to the parametric statistical tests; however, the small sample size and the non-characterization of the sample was a major limitation.

## 6. Conclusions

Our study demonstrated that in comparison with endurance exercise under NORM, endurance exercise under HYP (526 mmHg, simulated 3000 m) (e.g., same HR during exercise) induced a decrease in exercise load, greater metabolic stress (e.g., decreased SpO_2_, VO_2_, and oxygen pulse and increased RER), and skeletal muscle oxygenation responses (e.g., decreased oxhb and StO_2_ and increased dxhb) in healthy men. However, endurance exercise under HYP at the same HR level did not affect cardiac function compared with that under NORM in healthy men.

## Data Availability

The data presented in this study are available upon request from the corresponding author.
